# Applying Iterative Student Feedback across Flipped Classroom and Flexible Teaching Approaches: Impact on Veterinary Students’ Learning Experience

**DOI:** 10.3390/ani14162335

**Published:** 2024-08-13

**Authors:** Arti A. Singh, Frances M. Shapter, Anne Bernard, Deanne J. Whitworth, Marnie G. Holt, Philip S. Waller, Stephanie L. Bond

**Affiliations:** 1 School of Veterinary Science, Faculty of Science, The University of Queensland, Gatton, QLD 4343, Australia; a.singh1@uq.edu.au (A.A.S.); f.shapter@uq.edu.au (F.M.S.); d.whitworth@uq.edu.au (D.J.W.); 2 Faculty of Science, The University of Queensland, Brisbane, QLD 4072, Australia; m.holt2@uq.edu.au; 3 QCIF Bioinformatics, Institute for Molecular Bioscience, The University of Queensland, Brisbane, QLD 4072, Australia; a.bernard@qcif.edu.au; 4 eLearning Innovations and Partnerships in Science and Engineering (eLIPSE), The University of Queensland, Brisbane, QLD 4072, Australia; p.waller@uq.edu.au

**Keywords:** blended learning, flipped classroom, veterinary medicine, veterinary science, anatomy, physiology

## Abstract

**Simple Summary:**

Iterative feedback from students using a research-supported, flipped classroom delivery within a foundational veterinary gastrointestinal anatomy and physiology course suggested a strong preference for change from flipped to flexible delivery. Integration of broader course feedback enhanced the student experience in the subsequent flexible delivery iteration. Feedback requested the following: that online teaching materials be made available earlier in the semester rather than progressively; the re-introduction of face-to-face lectures; and full-length lecture videos with a more conversational presentation style in addition to already provided shorter, modularized videos. Once the enhanced flexible learning approach was employed, a substantially improved student experience was reported, with enhanced work/study/life balance, reduced stress levels, and more efficient time use when studying. Our results highlight the importance of considering student perceptions and the student experience when designing and implementing educational interventions. This teaching method offers veterinary educators an innovative and efficient starting point for designing foundational coursework.

**Abstract:**

No single teaching strategy supports all learning styles in veterinary science students. To facilitate more convenient and flexible teaching, learning, and revision, an innovative online digital learning platform—VetCloud—was developed to provide access to modularized programme content across courses to promote active, integrated learning. This study aimed to understand student perceptions regarding the enhancement of the student learning experience in a foundational course in gastrointestinal anatomy and physiology at The University of Queensland across two learning cycles, via applying iterative student feedback in transitioning a flipped classroom approach using VetCloud for the delivery of lecture content in 2022 to a flexible learning approach in 2023. By 2023, the use of VetCloud in the flexible learning approach improved students’ work/study/life balance, reduced their stress levels, and enabled a more efficient use of their time when studying, compared to the flipped classroom approach in 2022. Surveying student perceptions was integral to maximizing their learning experience. Data clearly demonstrates that students will mix-and-match how they interact with available options provided via flexible delivery on an individualized basis. This teaching method offers veterinary educators an innovative and efficient approach to veterinary student education in anatomy and physiology while enhancing student well-being.

## 1. Introduction

Higher education has undergone a sector-wide shift in recent years, away from largely face-to-face classroom-based instruction led by the ‘teacher as expert’ towards flexible, multi-modal instruction and an increased recognition and engagement of ‘students as partners’ [1] in the teaching and learning process. While emergency response teaching [2] at the time of the COVID-19 pandemic necessitated a rapid, large-scale online adaptation of teaching and assessment strategies [3], the advantages and benefits of eLearning approaches [4,5,6,7] such as blended learning and the flipped classroom in higher education have been highlighted across disciplines, including anatomy and physiology, medicine, veterinary science, and other health professions [8,9,10,11,12,13,14,15,16,17,18,19].

In the context of veterinary education, the use of blended learning and flipped classroom approaches has been reported to benefit the student learning experience in classroom settings [12,13,14], practical laboratory settings [15], and clinical and professional skills contexts [16,17,18]. Benefits to teaching staff have also been reported [12], including increased satisfaction in their teaching. Veterinary science students face the challenge of comprehending vast anatomical, physiological, and pathological variations between and across different animal species within a finite curriculum limited by programme length. Challenges associated with implementing the flipped classroom approach in veterinary education [12] include student-related barriers such as resistance to change and a perceived increase in an already heavy student workload, lack of engagement, and lack of time; staff-related barriers such as an increase in workload and lack of time for preparation of eLearning resources; and institutional barriers such as the availability of technology and resources, logistics, training, and institutional support.

The Bachelor of Veterinary Science (Honours) degree (BVSc(Hons)) at The University of Queensland (Australia) is a five-year undergraduate programme based at the University’s regional campus in Gatton, Queensland. The first four years of the degree involve a combination of lectures, tutorials, practical classes, and placements. Content is delivered and managed through the University’s online learning management system (LMS), Blackboard™ (by Anthology, Boca Raton, FL, USA). LMS course sites for previously completed courses are available to BVSc(Hons) students as they progress through their degree. The final year of the degree consists of internal and external clinical placements. The BVSc(Hons) students are a diverse cohort, ranging in age from 17 to 50+ years and with a variety of living arrangements, work demands, caring responsibilities, and financial constraints that may affect their ability to participate in on-campus or synchronous online classes in their pre-clinical years.

In an effort to facilitate more convenient and flexible teaching, learning, and revision for BVSc(Hons) students across their degree, VetCloud—an innovative online digital learning platform—was conceived and developed as a cross-faculty collaboration, providing students with access to modularized programme content across courses to enhance search capability and promote active, integrated learning. In VetCloud-using courses, lectures are designed using a scaffolding approach, where content is broken down into modules based on single knowledge topics, each with clearly defined learning outcomes. Lecture content for these courses is presented on VetCloud both as a complete full-length recording and as short modular videos (3–12 min), accompanied by downloadable learning resources including lecture slides, notes, slides with an accompanying transcript, and extension resources. Active learning strategies are incorporated to maximize student engagement via the use of self-assessment quiz questions associated with each module provided on VetCloud. This enables students to learn at their own pace, consolidating knowledge and understanding, and easily locate material on VetCloud for revision of ‘assumed knowledge’ in later years of the programme. Iterative student feedback has informed enhancements to learning materials provided on VetCloud each active learning cycle, culminating in quantifiably improved student impact.

Gathering and responding to student feedback has been central to the iterative development of VetCloud to date. An important source of student feedback has been partnerships between VetCloud-using courses and the Student-Led Observation for Course Improvement (SLOCI) [20] Team within the Faculty of Science in 2022 and 2023. The Science SLOCI Team is a student-led team of current undergraduate students enrolled in programmes from across the Faculty of Science at The University of Queensland who work to gather information regarding current student perceptions of courses or projects on behalf of teaching staff. Based on the ‘students as partners’ philosophy [1], SLOCI aims to deliver the student voice to teaching staff in more targeted ways than institutional end-of-semester teaching evaluations allow, through a combination of tailored activities such as online surveys, focus group interviews, and class observations.

VETS1003: Digestion, Metabolism and Nutrition is a foundational one-semester gastrointestinal anatomy and physiology course for BVSc(Hons) students. Conducted in semester 2 each year, VETS1003 is taken by first-year BVSc(Hons) students in the second semester of their degree programme. VetCloud has been used in VETS1003 in two learning cycles to date: semester 2, 2022 and semester 2, 2023. The aim of this study was to enhance the student learning experience in VETS1003 via iterative feedback, based on the two learning cycles of first-year BVSc(Hons) student feedback obtained from online surveys facilitated through partnerships with the Faculty of Science SLOCI Team (i.e., from feedback obtained from the 2022 and 2023 cohorts of VETS1003 students).

## 2. Materials and Methods

VetCloud was used to deploy the anatomy and physiology lecture content during weeks 1–9 of the 13-week teaching semester in the first-year course VETS1003: Digestion, Metabolism and Nutrition in semester 2, 2022 (*n* = 145 students) and 2023 (*n* = 133 students).

### 2.1. Active Learning Cycle 1: VetCloud Used in a Flipped Classroom Approach

In semester 2, 2022, VETS1003 learning resources were provided to students via a flipped classroom approach on VetCloud, with progressive weekly release of lecture content just prior to timetabled ‘lecture’ timeslots (3 h per week) in weeks 1–9. Content was subsequently discussed during hybrid tutorials held concurrently face-to-face and online via Zoom^TM^ (San Jose, CA, USA), and then applied in practical classes. The ‘complete lecture recording videos’ provided in 2022 were not recordings of live lectures; they consisted of the relevant pre-recorded short topic videos joined together to cover all the topics relevant to each lecture within a single longer video.

### 2.2. Active Learning Cycle 2: VetCloud Used in a Flexible Learning Approach

In semester 2, 2023, all content for the anatomy and physiology modules of VETS1003 was provided on VetCloud from week 1 of the semester. In addition to VetCloud learning resources, face-to-face lectures (3 h per week) were offered in weeks 1–7 of the 13-week teaching semester. In 2023, the ‘complete lecture recording videos’ available in 2022 that consisted of the relevant short topic videos joined together were provided to students from the beginning of the semester (i.e., before the face-to-face lectures took place) and were replaced with a recording of each 2023 face-to-face lecture within one week of each lecture being delivered during the semester. Content was subsequently discussed during weekly face-to-face tutorials, then applied in practical classes.

### 2.3. Student Surveys

This study was approved by the Human Ethics Committee of The University of Queensland (project number 2019/HE001666). The SLOCI Team conducted online voluntary, anonymous surveys of VETS1003 students during week 8 of semester 2 in 2022 (Supplementary File S1) and weeks 6–9 of semester 2 in 2023 (Supplementary File S2) via the Microsoft Forms platform. VETS1003 students were informed about the surveys and invited to participate in them via weekly announcements made on the VETS1003 LMS course sites and sent via email. Implied consent was obtained from all students involved in the study by their voluntary choice to respond to a survey, and students were advised that survey responses would remain anonymous to VETS1003 teaching team members. Students were given the incentive of winning one of a limited number of AUD 20 Coles-Myer retail gift cards for their survey participation. Both the 2022 and 2023 surveys included two additional, voluntary open-response questions, through which students who wished to participate in the gift card draw could provide their names and contact email addresses. If provided, student names and contact details were used only for gift card draws.

The 2022 online survey (Supplementary File S1) consisted of 33 questions, including 11 Likert scale rating questions, 14 multiple-choice questions that all allowed for one response each, and eight open-response questions. Survey questions were refined for the 2023 online survey (Supplementary File S2) to obtain more nuanced information about how students interacted with VetCloud; the 2023 survey consisted of 32 questions, including nine Likert scale rating questions, seven multiple-choice questions that allowed for only one response each, six multiple-choice questions that allowed for multiple responses, and ten open-response questions.

### 2.4. Statistical Methods

Descriptive statistics were reported using frequencies and proportions for categorical data. Likert scales were considered in two ways: categorical, with five categories (1, 2, 3, 4, and 5), and ordinal (scale from 1 to 5), which were described using medians and inter-quartile ranges (IQR). Comparison of categorical variables between learning cycles (2023 vs. 2022) was performed using a chi-square test. Comparison of the ordinal Likert scales between the learning cycles was performed using a Mann–Whitney test. In figures, data from the 2022 learning cycle are presented in blue and those for 2023 are displayed in red. All analyses were performed using the R statistical software [21], and *p*-values were two-tailed with *p* < 0.05 considered statistically significant.

## 3. Results

VETS1003 student perceptions of VetCloud were compared between the implementation of a flipped classroom approach in 2022 (*n* = 47 students survey responses, 32% response rate) and a flexible learning approach in 2023 (*n* = 46 student survey responses, 35% response rate).

### 3.1. Students’ Lecture Delivery Format Preferences during the Flipped Classroom and Flexible Learning Approaches

**Figure 1:**
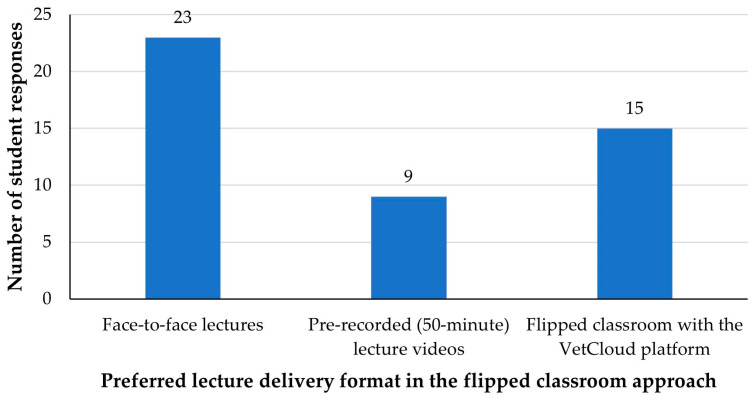
VETS1003 students’ (*n* = 47) preferred lecture delivery formats during the flipped classroom approach employed in 2022. Face-to-face lectures and pre-recorded (50 min) lecture videos were not offered in 2022, and students were permitted to select only one of the three answer options.

**Figure 2:**
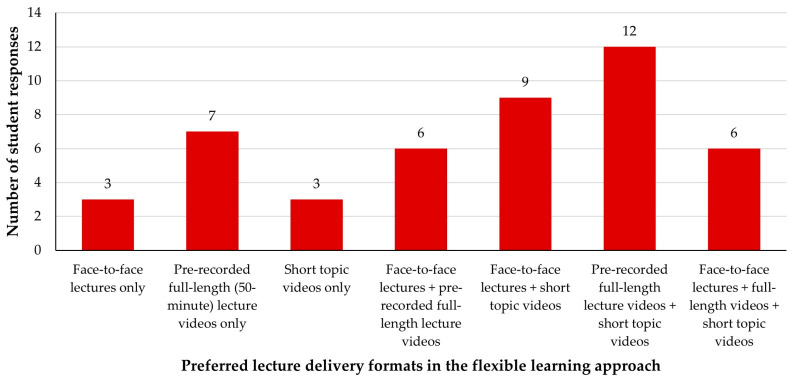
VETS1003 students’ (*n* = 46) preferred lecture delivery formats during the flexible learning approach employed in 2023. Students were permitted to select as many of the three answer options as they wished.

Due to COVID-19 and resultant staffing changes in 2021, the previous two VETS1003 student cohorts (2020 and 2021) had been provided with lecture recordings through the LMS when face-to-face lectures were not possible. In 2022, student preferences were assessed regarding a return to face-to-face lectures, recordings of previously delivered live lectures, or a flipped classroom approach with the VetCloud platform (Figure 1). Given the clear student preference for a return to the live lecture (Figure 1) when they were permitted to select only one favored lecture delivery format, live lectures were incorporated in 2023 and the survey questions were adjusted accordingly (Figure 2).

There was a clear student preference for a multi-modal delivery of content in 2023, with the majority of students (*n* = 33/46, 72%) selecting multiple formats (Figure 2) now that they were allowed to do so. Thirteen students (28%) selected only one lecture format, of which pre-recorded full-length (50 min) lecture videos were the most popular (*n* = 7 students, 15%). Overall, 31 of the 46 students (67%) reported a liking for pre-recorded full-length (50 min) lecture videos, 30 students (65%) a liking for short topic videos, and 24 students (52%) a liking for face-to-face lectures.

### 3.2. Timeline of Student Engagement with Resources on VetCloud during the Flipped Classroom and Flexible Learning Approaches

**Figure 3:**
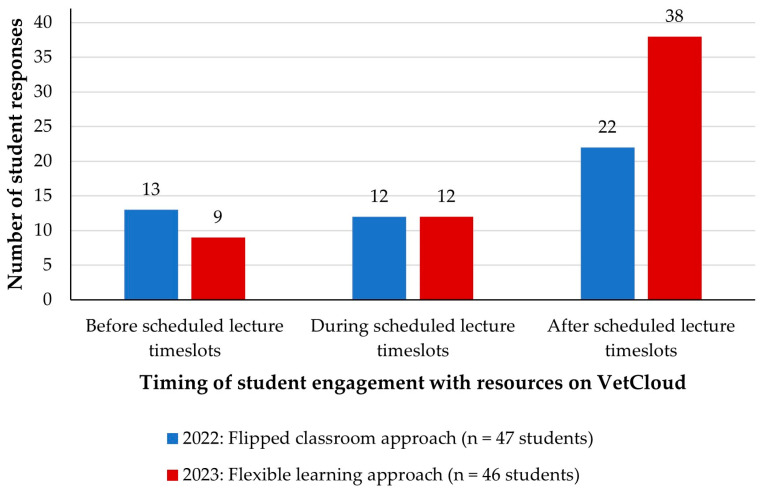
Timing of VETS1003 students’ engagement with resources on VetCloud in relation to scheduled ‘lecture’ timeslots during the flipped classroom approach employed in 2022 and the scheduled face-to-face lectures during the flexible learning approach employed in 2023. Students in 2022 were permitted to select only one of the three answer options, while students in 2023 were permitted to select as many of the three answer options as they wished.

In 2022, 28% of students (*n* = 13/47) engaged with the learning resources on VetCloud for weekly ‘lectures’ prior to the scheduled self-directed learning ‘lecture time’, while 25% (*n* = 12) interacted with the resources during the ‘lecture time’ (Figure 3). Almost half of the students (*n* = 22, 47%) engaged with the learning resources after the scheduled ‘lecture time’ (Figure 3).

**Figure 4:**
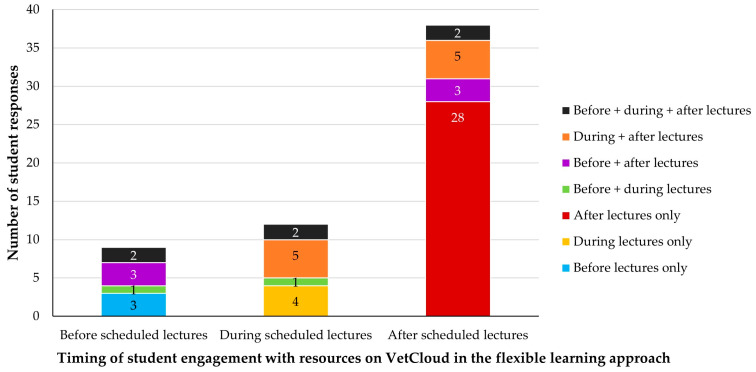
Timing of VETS1003 students’ (*n* = 46) engagement with resources on VetCloud in relation to the scheduled face-to-face lectures during the flexible learning approach employed in 2023, showing the specific combinations of timing options they selected.

In 2023, when face-to-face VETS1003 lectures were held, the learning resources on VetCloud were available to students from the beginning of the semester and multiple responses to the equivalent survey question were allowed. The 46 responding students provided 59 responses (Figure 3). Eleven students (24%) selected multiple answer options, including two (4%) who selected all three times, with 35 students (76%) indicating that they only engaged with VetCloud resources at one time (Figure 4). Nine students (20%) engaged with the resources on VetCloud before the face-to-face lectures, 12 students (26%) engaged with them during the lectures, and 38 (83%) engaged with them afterwards (Figure 4). Of the 38 students (83%) who engaged with the resources after the face-to-face lectures, 28 (61% of the total 46 survey respondents) engaged with the resources only after the lectures, three students (7%) engaged with them both before and after the lectures, and five students (11%) engaged with them both during and after the lectures (Figure 4).

A chi-square test was performed to compare the timing of engagement with resources on VetCloud between the two learning cycles. For the purpose of the analysis, all responses by 2023 students were considered (*n* = 59 responses). No significant relationship was found (*p* = 0.16).

### 3.3. Students’ Tutorial Attendance and Engagement during the Flipped Classroom Approach

**Figure 5:**
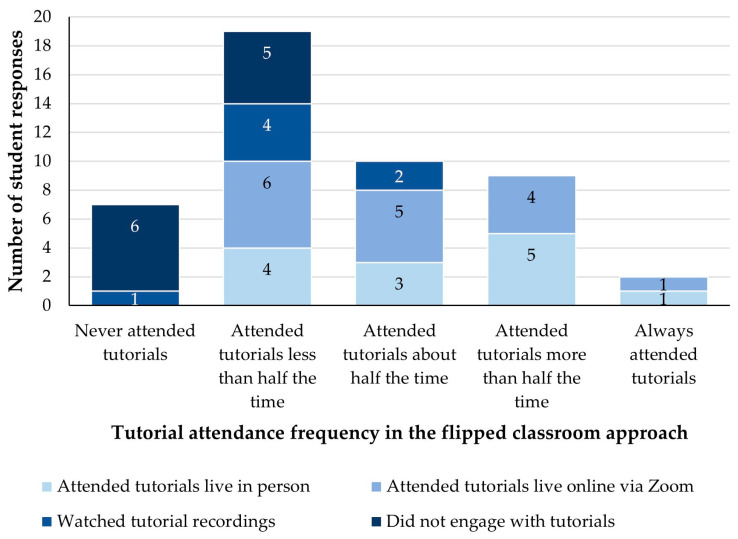
The reported frequency of tutorial attendance and mode of tutorial engagement of VETS1003 students (*n* = 47) during the flipped classroom approach employed in 2022.

In 2022, when face-to-face lectures were not held for VETS1003 and tutorials were held in-person and synchronously online via Zoom^TM^, students were asked about their tutorial attendance and mode of engagement. They were first asked to respond to the Likert scale rating question ‘*How often do you attend the tutorial sessions, either in person or over Zoom?*’. After this, they were asked to respond to the multiple-choice question ‘*How do you generally engage with the weekly tutorial?*’ by selecting one answer option (Figure 5). Students who selected the ‘*I do not engage with tutorials*’ answer option were then directed to an open-response question that asked them to explain why this was the case.

The majority of students (*n* = 26/47, 55%) indicated that they attended tutorials less than half of the time or did not attend them at all (Figure 5). Ten students (21%) attended approximately half of the tutorial sessions, nine students (19%) attended more than half of the tutorial sessions, and two students (4%) indicated that they always attended the tutorials (Figure 5).

Of the 19 students who indicated that they attended less than half of the tutorials, six (13% of the responding 47 students) typically did so online via Zoom and four (9%) in person (Figure 5). Another four (9%) of these students indicated that they typically watched video recordings of the tutorials, as did one (2%) of the seven students who never attended tutorials. The remaining five students (11%) who attended less than half the tutorials and six students (13%) who never attended tutorials reiterated that they did not typically engage with tutorials at all (Figure 5).

### 3.4. Time Spent Engaging with Resources on VetCloud during the Flipped Classroom and Flexible Learning Approaches

**Figure 6:**
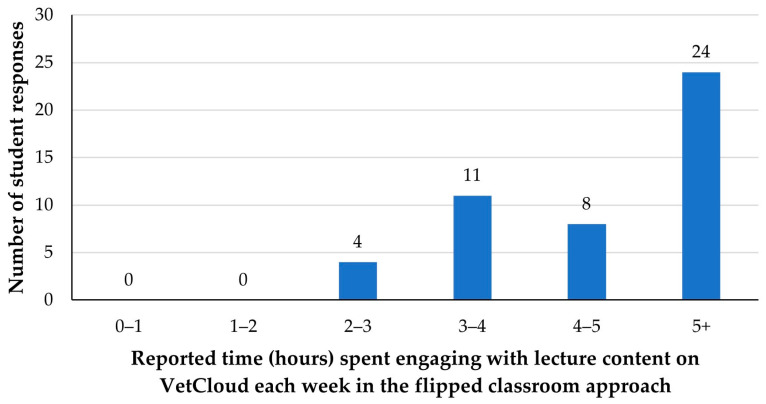
The average amount of time VETS1003 students (*n* = 47) reportedly engaged with lecture content/resources on VetCloud each week during the flipped classroom approach employed in 2022.

In 2022, all 47 responding students reportedly engaged with the lecture content for an average of at least two hours each week, with just over half the students (*n* = 24, 51%) indicating that they spent at least five hours engaging with lecture content each week (Figure 6).

**Figure 7:**
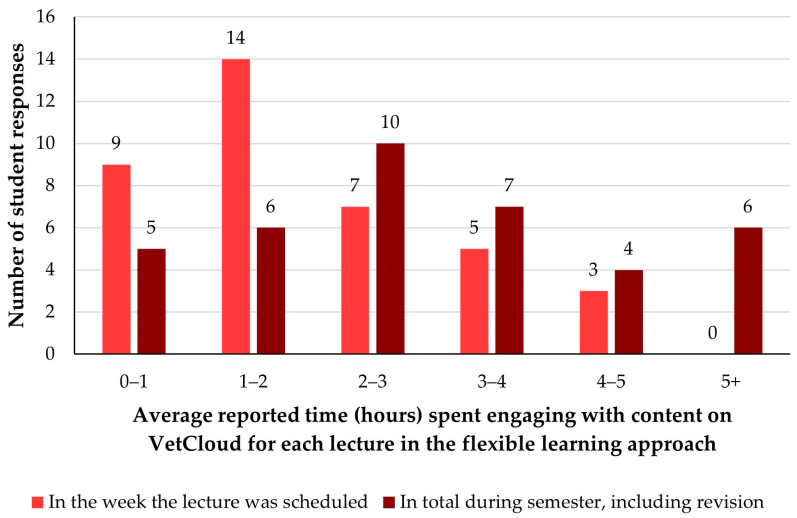
The average amount of time VETS1003 students (*n* = 38) reportedly spent engaging with content/resources on VetCloud for each lecture during the flexible learning approach employed in 2023, in the week each lecture was scheduled and in total during the semester including for revision.

The broader multiple-choice question ‘*On average, how many hours do you engage with the lecture content a week?*’ from the 2022 survey was replaced in the 2023 survey with two different multiple-choice questions aimed at teasing out more nuanced information about when and how students were spending time engaging with VETS1003 resources on VetCloud for **each** lecture. In 2023, students were first asked ‘*On average, for how many hours do you engage with the content for **each** lecture on VetCloud in the week that it is timetabled?*’ and then ‘*On average, for how many hours do you engage with the content for **each** lecture on VetCloud in total, including revision for assessments?*’ (Figure 7).

Of the 46 survey respondents in 2023, eight selected a larger number of hours per lecture in the week that it was timetabled rather than in total during the semester; the responses to these two questions from these eight students have not been included in our analysis, and thus 38 responses for each of these two questions are reported and analysed here instead of 46 responses each. These 38 students spent a reported average of five hours or less engaging with content for each lecture in the week it was scheduled (Figure 7), most commonly (*n* = 14/38, 37%) spending 1–2 h on each lecture. In terms of total time spent engaging with content for each lecture during the semester including for revision, students most commonly (*n* = 10/38, 26%) indicated that they spent an average of 2–3 h engaging with the content for each lecture, followed closely by students (*n* = 7/38, 18%) who spent 3–4 h (Figure 7).

**Figure 8:**
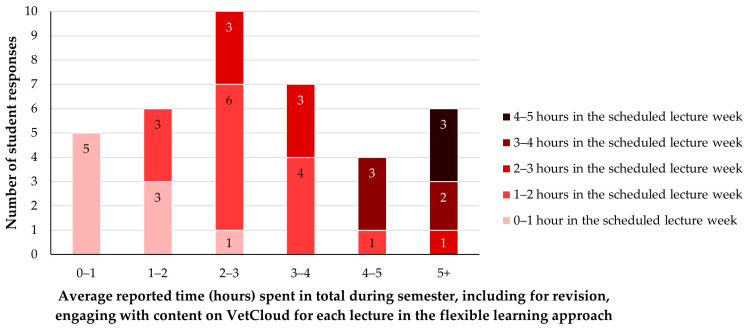
The average amount of time VETS1003 students (*n* = 38) reportedly spent engaging with content/resources on VetCloud for each lecture during the flexible learning approach employed in 2023, showing the number of hours each student spent during the week each lecture was scheduled out of the total number of hours they spent during the semester.

Eleven students (29% of 38) indicated that they engaged with lecture content/resources on VetCloud only in the week when each lecture was scheduled (Figure 8). The majority of the 38 students (*n* = 27, 71%) indicated that they did engage with lecture content/resources on VetCloud after the week when each lecture was scheduled; the largest proportion (*n* = 6, 16%) engaged with it for 1–2 h per lecture in the week each lecture was scheduled out of a total of 2–3 h per lecture over the semester, followed by four students (~11%) who engaged with it for 1–2 h per lecture in each scheduled lecture week out of a total of 3–4 h per lecture over the semester (Figure 8). Most students (*n* = 18/38, 47%) indicated that they spent an additional 1 h per lecture for revision over the semester (Figure 8).

### 3.5. Student Opinions of Whether Sufficient Learning Materials Were Provided on VetCloud to Support Their Learning as Part of the Flipped Classroom and Flexible Learning Approaches

**Figure 9:**
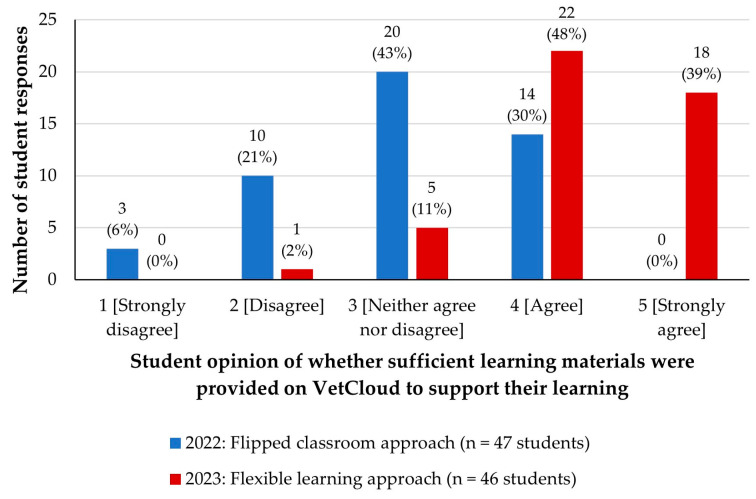
VETS1003 students’ opinions of whether sufficient learning materials were provided on VetCloud to support their learning as part of the flipped classroom approach employed in 2022 (*n* = 47) and the flexible learning approach employed in 2023 (*n* = 46).

In 2022, students were asked the multiple-choice question ‘*Do you feel that the flipped classroom approach has provided sufficient learning materials to give you confidence that you understand the content, as outlined by the learning outcomes for each module?*’ and were directed to select one answer option (Figure 9).

In 2023, after the flexible learning approach, with the online content available from the beginning of the semester, the multiple-choice question from the 2022 survey was replaced with a more clearly worded question that asked students to rate on a Likert scale of 1–5 how much they agreed with the statement ‘*VetCloud provides access to sufficient learning materials to support my learning in VETS1003*’.

**Figure 10:**
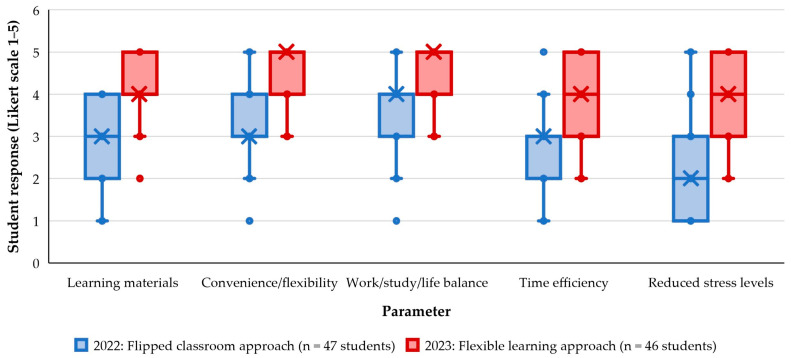
VETS1003 students’ evaluations of VetCloud’s impact on their learning experience; that is, whether it provided sufficient learning materials to support their learning, provided convenience and flexibility in their learning, supported their work/study/life balance, enabled them to use their time more efficiently when learning, and helped to reduce their stress levels as part of the flipped classroom approach employed in 2022 (*n* = 47) and the flexible learning approach employed in 2023 (*n* = 46). (× = median; lines = quartiles, median, and minimum and maximum values; dots = Likert scale answer values, including outliers).

A chi-square test was performed to assess the relationship between student opinion and the learning cycle, and a significant difference was found (*p* < 0.001). In 2023, the majority (87%) of students agreed (48%) or strongly agreed (39%; Figure 9), compared to only 30% who agreed in 2022. The calculated median Likert rating value for the 2023 cohort was 4 (IQR 4–5), compared to the 2022 cohort’s calculated median rating value of 3 (IQR 2–4; Figure 10). A Mann–Whitney test was performed to assess the difference between the two learning cycles and the result was significant (*p* < 0.001).

### 3.6. Student Evaluations of Whether Having Access to VetCloud as Part of Flipped Classroom and Flexible Learning Approaches Provided Greater Convenience and Flexibility in Their Learning

**Figure 11:**
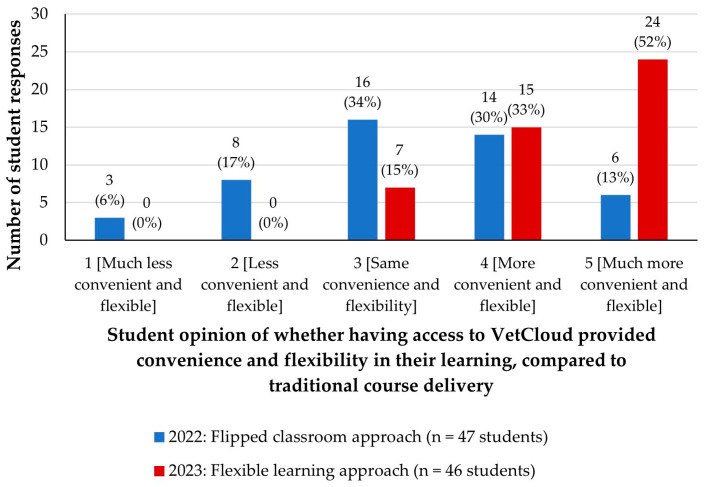
VETS1003 students’ opinions of whether having access to VetCloud provided convenience and flexibility in their learning in comparison to traditional course delivery during the flipped classroom approach employed in 2022 (*n* = 47) and the flexible learning approach employed in 2023 (*n* = 46).

In 2022, students were asked to rate how they found the flipped classroom approach using the VetCloud platform for providing convenience and flexibility in their learning in comparison to traditional course delivery (didactic lectures; Figure 11). In 2023 (flexible learning approach), students were asked ‘*On a scale of 1–5, how do you rate the VetCloud platform for providing convenience and flexibility in your learning, in comparison to traditional course delivery (didactic lectures delivered at set times)?*’ (Figure 11).

In 2022, when a flipped classroom approach was employed with online resources provided progressively over the semester in a ‘just-in-time’ manner, students provided a spread of responses across all five Likert scale levels, with the greatest proportion of them selecting a rating of 3 (same convenience and flexibility; *n* = 16, 34%; Figure 11) or 4 (more convenient and flexible; *n* = 14, 30%), with a calculated median rating value of 3 and IQR 3–4 (Figure 10). In 2023, all students selected a rating of 3–5 (calculated median rating 5 and IQR 4–5), with more than half of the students (*n* = 24, 52%) reporting that having access to VetCloud resources provided more convenience and flexibility in their learning, in comparison to traditional course delivery (Figure 10 and Figure 11). A chi-square test was performed on the categorical data to assess the relationship between students’ rating and learning cycle, and a significant increase in ratings was found for 2023 (*p* < 0.001), with 85% of students selecting a rating of 4 or 5 in 2023, compared to 43% in 2022. Similarly, a Mann–Whitney test was performed on the Likert scale data, which provided a similar interpretation (*p* < 0.001). 

### 3.7. VetCloud’s Facilitation of Students’ Work/Study/Life Balance during the Flipped Classroom and Flexible Learning Approaches

**Figure 12:**
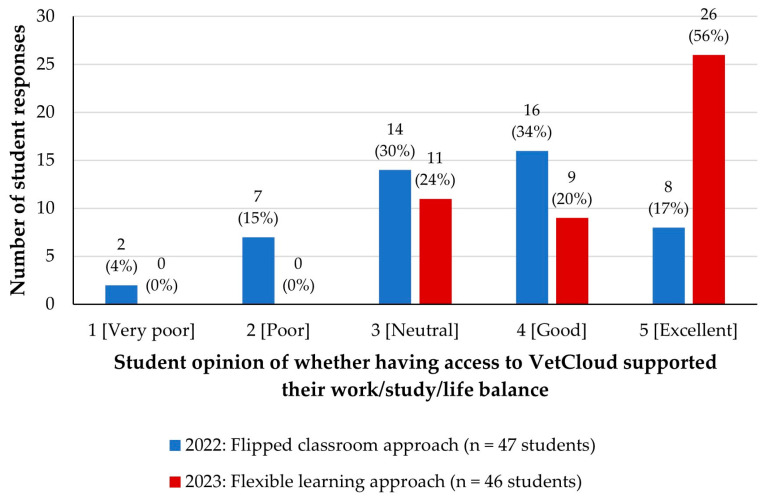
VETS1003 students’ opinions of whether having access to VetCloud supported their work/study/life balance as part of the flipped classroom approach employed in 2022 (*n* = 47) and the flexible learning approach employed in 2023 (*n* = 46).

Students were also asked to rate the VetCloud platform in terms of how well it supported their work/study/life balance. In 2022, they provided a spread of responses across all five Likert scale levels (Figure 12), with 81% rating VetCloud at 3–5 (neutral–excellent; Figure 11). In 2023, all 46 survey respondents rated VetCloud at 3–5 (neutral–excellent; Figure 12). Both a chi-square test on the categorical data and a Mann–Whitney test on the Likert scale data presented evidence for a significant increase in ratings from 2022 to 2023 (*p* < 0.001 for both). The calculated median Likert scale rating was 5 (IQR 4–5) in 2023, whereas it had been 4 (IQR 3–4) in 2022. Figure 10 shows the increase in the calculated median ratings between the 2022 (4, IQR 3–4) and 2023 (5, IQR 4–5) cohorts.

### 3.8. VetCloud’s Facilitation of Efficient Time Use in the Flipped Classroom and Flexible Learning Approaches

**Figure 13:**
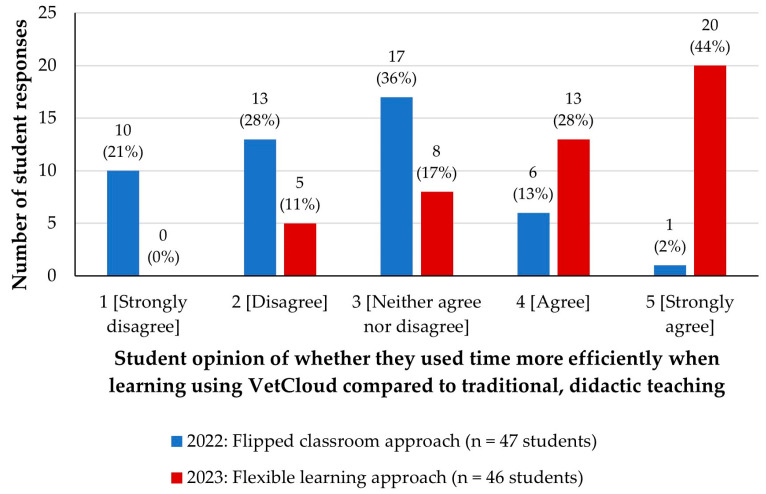
VETS1003 students’ opinions of whether they used their time more efficiently when learning using VetCloud compared to traditional, didactic teaching as part of the flipped classroom approach employed in 2022 (*n* = 47) and the flexible learning approach employed in 2023 (*n* = 46).

In 2022, with the progressive release of the online content and no recordings of live lectures provided, students were asked ‘*Do you feel that you are using your time more efficiently learning in this format, compared to didactic teaching (traditional lectures)?*’. Cumulatively, 85% of students were neutral to being strongly in disagreement with this statement (Figure 13).

In 2023, when the online content was available from the beginning of the semester, the multiple-choice question from the 2022 survey was replaced with a more clearly worded question that asked students to rate how much they agreed with the statement ‘*I use my time more efficiently when learning using VetCloud, compared to didactic teaching (traditional lectures)*’. This time, most students (Figure 13) either strongly agreed (*n* = 20/46, 44%) or agreed (*n* = 13, 28%) with the given statement. The calculated median rating value for this 2023 survey question was 4 (IQR 3–5)—a marked increase from the equivalent 2022 median rating value of 3 (IQR 2–3; Figure 10). Both a chi-square test on the categorical data and a Mann–Whitney test on the Likert scale data presented evidence for a significant increase in rating from 2022 to 2023 (*p* < 0.001).

### 3.9. Student Stress Levels during the Flipped Classroom and Flexible Learning Approaches

**Figure 14:**
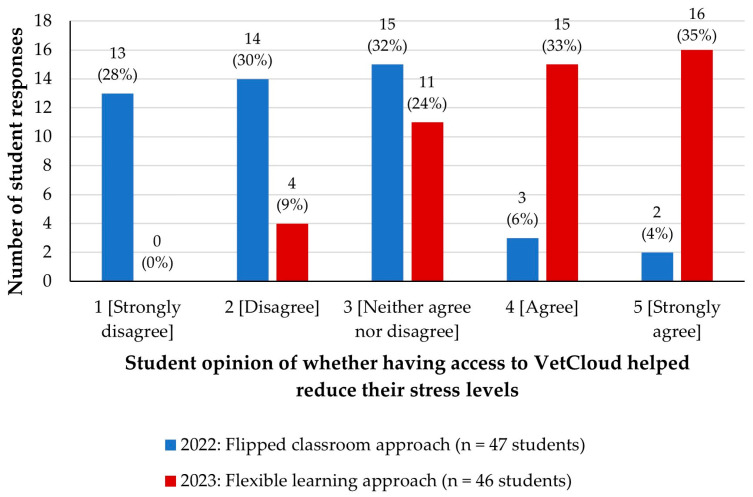
VETS1003 students’ opinions of whether having access to VetCloud as part of the flipped classroom approach employed in 2022 (*n* = 47) and the flexible learning approach employed in 2023 (*n* = 46) helped to reduce their stress levels during the semester.

In the 2022 survey, students were asked ‘*Do you feel the flipped classroom approach using VetCloud has reduced your stress levels while learning the content throughout the semester?*’. Most students (*n* = 42/47, 89%) were neutral, disagreed, or strongly disagreed (Figure 14).

In 2023, the multiple-choice question from the 2022 survey was replaced with a question that asked students to rate how much they agreed with the statement ‘*Having access to VetCloud as a learning resource has reduced/is reducing my stress levels during the semester, including in the lead up to assessments*’. This time, with all the VETS1003 anatomy and physiology content provided on VetCloud from the beginning of the semester as part of the flexible learning approach, there was a positive trend, with most students either strongly agreeing (*n* = 16/46, 35%) or agreeing (*n* = 15, 33%) with the given statement (Figure 14). The calculated median rating value for this 2023 survey question was 4 (IQR 3–5) compared to 2 (IQR 1–3) in 2022 (Figure 10). Both a chi-square test on the categorical data and a Mann–Whitney test on the Likert scale data presented evidence for a significant increase in rating from 2022 to 2023 (*p* < 0.001).

### 3.10. Students’ General Opinions of the Flipped Classroom and Flexible Learning Approaches

In the 2022 survey, students were asked three final open-response questions to gauge their general opinions about the flipped classroom approach. They were first asked ‘*What did you like/dislike about the VetCloud learning platform? Are there any improvements that you would suggest?*’ (*n* = 40/47 responses, 85%) and finally ‘*Do you have any further comments, or feedback on the course thus far?*’ (*n* = 28 responses, 60%). Across these three questions, students indicated that they appreciated several of the resources provided on VetCloud (e.g., self-assessment quizzes, short topic videos) and generally found the platform itself easy to navigate and use. However, students commonly felt that the use of VetCloud in the flipped classroom approach could be improved. Students did not like the restricted release of online content/resources on Thursdays before being expected to engage with it on Friday mornings (8–11 a.m.) in preparation for attending tutorials (12–1 p.m.) and practical classes (1–5 p.m.) on Friday afternoons. They found the same-day engagement/delivery of lecture content, tutorials, and practicals too overwhelming and fast-paced, and reiterated that it did not provide them enough time to ‘digest’ the information.

In 2022, students felt that there was too much lecture content and found the time allocated for engaging with it on Friday mornings unrealistic and stressful: ‘*the course is so content heavy that it is difficult to get the lectures done in the 3 hours before the prac on the Friday. And if I do get the lectures done, I don’t have a good idea of the content going into the prac that day.*’ Several students felt that the delivery of content in the short topic videos was too fast, which made the concepts covered more difficult to understand: ‘*I think VETS1003 is very detailed and complicated and therefore it requires more time and slower paced, in depth lectures.*’ Students requested slower, more in-depth explanations of complicated concepts in videos delivered in a more conversational style, and earlier release of online content/resources on VetCloud before scheduled classes. Several students also expressed a preference for having—and requested the addition of—face-to-face lectures for VETS1003, to allow for more conversational and in-depth explanations and to provide greater opportunities for asking questions, clarifying concepts, and interacting with their peers and teaching staff.

In the 2023 survey, students were similarly asked four final open-response questions to gauge their general opinions about the flexible learning approach. The questions asked were ‘*What do you like about the VetCloud platform? Please explain.*’ (*n* = 46/46 responses), ‘*What do you dislike about the VetCloud platform? Do you have any suggestions for improvement? Please explain.*’ (*n* = 20 responses, 43%), and ‘*Do you have any other comments about the VetCloud platform and/or how it has been used in VETS1003?*’ (*n* = 9 responses, 20%). Students again praised the ease of use of the VetCloud platform and appreciated the provided resources, with suggestions for improvement largely based around technical aspects (e.g., requests for a greater variety of video playback controls) or requests for further resources (e.g., more quiz questions). Students liked having the option to attend face-to-face lectures, having access to video recordings of live lectures available for later viewing on VetCloud soon afterwards, and particularly having the other provided resources (short topic videos, learning outcomes, lecture slides, lecture slides with transcript notes, and self-assessment quizzes) available to them on VetCloud even before face-to-face lectures were held. Students felt that these factors allowed them greater flexibility in their learning, provided greater access to lecture content without the absolute requirement for commuting to campus, and positioned VetCloud as a one-stop ‘hub’ for learning resources that suit students’ different learning preferences.

## 4. Discussion

There was a marked improvement in how well VetCloud supported students’ work/study/life balance from 2022 to 2023, with the majority (56%) of students in 2023 rating the platform as excellent. Students in 2023 reported that they used their time much more efficiently when studying using VetCloud in the flexible learning approach, than the students in the flipped classroom approach in 2022. Similarly, students in 2023 also found that being able to use VetCloud in the flexible learning approach reduced their stress levels to a greater extent than the students in the flipped classroom approach in 2022.

It is an acknowledged limitation of this study that we are unable to compare student data from multiple learning cycles of VETS1003 that differed solely by one intervention (e.g., two iterations of a flipped classroom approach that included only changes to the online resources provided or our 2022 flipped classroom approach and a flexible learning approach that introduced face-to-face lectures without any other changes). However, this is active scholarship of teaching and learning (SoTL) research embedded in courses delivered in real time; fairness and justice in the impact of educational interventions on students is of paramount importance [22], and it is not ethical to repeat the recognized mistakes or limitations of a previous learning cycle and restrict student opportunities and/or benefits for the sake of having a repeatable study. The most ethical approach for us to take was to act on the clear, constructive student feedback from 2022 to improve the student learning experience into the next year rather than focusing on one single intervention, which resulted in the flexible learning approach we employed in 2023. Hence, our study is focused on the impacts of our employed flipped classroom and flexible learning approaches in two consecutive learning cycles on student experience from student perception data and feedback from 2022 and 2023. The incentive provided to students for survey participation—the opportunity to win one of a limited number of AUD 20 Coles-Myer retail gift cards if they identified themselves—potentially introduced enrolment bias into this study. Students were informed that all survey responses would remain anonymous to VETS1003 teaching staff, and that providing names and contact details with their otherwise-anonymous survey responses was strictly voluntary and only for gift card draw purposes. However, the perceived potential for survey responses to be identified could have resulted in some students declining to participate.

There is no single tool that meets all the curriculum requirements for teaching veterinary anatomy and physiology; every student has a unique learning style. This is consistent with an overall improvement in assessed parameters associated with the 2023 multi-modal presentation of content including online resources as well as face-to-face lectures—provision of resources in a format that offered students choice. Positive effects on both student performance and well-being of medical students using digital learning resources for self-directed study have been demonstrated in multiple disciplines, including anatomy [23], radiology [24], histology [25], ECG interpretation [26], and ultrasound training [27]. There is a significant positive relationship between psychological characteristics and academic fit in veterinary students [28]. Veterinary students can experience high levels of anxiety, depression, and decreased overall life satisfaction [29,30,31,32]; there is a positive relationship between psychological characteristics and engagement, and a negative relationship with exhaustion [28]. VetCloud is a practical intervention that supports the academic experience of veterinary students by addressing both their academic needs—by 2023 students felt that VetCloud provided them with sufficient learning materials to support their learning—and importantly also their psychological needs. Use of VetCloud in the flexible learning approach in 2023 improved students’ work/study/life balance, reduced their stress levels, and enabled a more efficient use of their study time, compared to the flipped classroom approach used in 2022. VetCloud therefore offers educators a more holistic educational tool to support veterinary education.

While active teaching and learning approaches such as the flipped classroom are generally reported to be beneficial, such teaching approaches have not been implemented widely in veterinary science education because of the inherent challenges posed by the integrated nature of the curriculum [12] and by consistently poor student feedback. While a variety of offline and online teaching resources and activities have been developed worldwide for teaching veterinary science in recent years [13,14,15,16,17,18,33,34,35,36,37,38,39,40], the manner in which they are used can vary significantly based on specific institutional, ethical, disciplinary, or other requirements—including barriers to the implementation of flipped classroom-type approaches [12]. Multiple small changes to teaching methods employed in tandem, as we employed in 2023, can thus be very beneficial for student experience, student efficiency, and learning quality. The importance of seeking and considering student perspectives when evaluating the efficacy of teaching methods in veterinary science [15,39,40] and medicine [23,24,41] has been highlighted.

In 2022, when face-to-face anatomy and physiology lectures were not held and no recordings of full-length live lectures from previous years were available for reference, 49% of students stated that they would have preferred to have lectures delivered face-to-face, indicating a solid demand for a return to face-to-face learning. However, almost one-quarter of students (23%) did not engage with tutorials at all, largely because they either had not been able to complete watching the relevant lecture content videos on VetCloud on Friday morning prior to the scheduled tutorial time on Friday afternoon or they had watched the relevant videos but had not had sufficient time to ‘digest’ the content. Students who answered open-ended general feedback questions in the 2022 survey clearly explained that they did not appreciate the late release of online content/resources on Thursdays and that they found the same-day engagement/delivery of lecture content, tutorials, and practicals on Fridays too overwhelming, fast-paced, and stressful. Constructively, students requested the earlier release of online content/resources on VetCloud before scheduled classes, alongside slower, more in-depth explanations of complicated concepts in videos delivered in a less formal and more conversational manner and the addition of face-to-face lectures.

Responding to this feedback from 2022 students, face-to-face lectures were introduced for the anatomy and physiology modules of VETS1003 in 2023 and they were scheduled on Monday mornings, prior to face-to-face tutorials on Tuesday afternoons and practical classes on Friday afternoons. As part of the new flexible learning approach, the resources on VetCloud were this time provided to students from week 1 of the semester, facilitated by the development of the bulk of these resources in 2022, instead of progressive weekly release. Additionally, complete lecture recording videos of the live lectures were made available on VetCloud within one week of each lecture being held in 2023. This response to student feedback was associated with a stronger preference for VetCloud resources as a lecture delivery format, with 67% of students reporting a liking for full-length (50 min) lecture videos and 65% of students liking the short topic videos provided on VetCloud. Students who answered open-response questions in the 2023 survey appreciated having the option to attend face-to-face lectures and/or watch the video recordings of these live lectures on VetCloud soon afterwards, and particularly appreciated having the other provided resources (short topic videos, learning outcomes, lecture slides, lecture slides with transcript notes, and self-assessment quizzes) available to them on VetCloud even before face-to-face lectures were held. Students largely enjoyed the greater flexibility in their learning and greater access to lecture content provided by the changes made to the timing and provision of resources on VetCloud and the scheduling of learning activities in going from the flipped classroom approach in 2022 to the flexible learning approach in 2023.

While lectures are an effective method of conveying information [42], a benefit of the VetCloud platform is the provision of both short topic videos and full-length (50 min) lecture recordings, which provides students with diverse learning styles the advantage of choice regarding how they would prefer to engage with core knowledge content. It has been argued that there is an inverse relationship between the length of a lecture and the retention of material covered due to ‘attention lapses’ [43], supporting the justification of shorter modularized content; although issues with the analysis undertaken preclude broader extrapolation of the data presented [42]. However, in the context of modern veterinary education, there are additional benefits regarding modularized delivery of content beyond the potential for improved retention of knowledge. Veterinary students are a highly diverse cohort, with students ranging in age from 17 to 50+ years, and many students are currently under significant financial constraints. Attendance at a regional campus can raise conflicts with work, children, and caring responsibilities. VetCloud supports learning in a way that influences, motivates, and inspires students to learn, while embracing and highlighting inclusivity, catering to the diverse learning styles and life situations of our students. Examples of this include anecdotal feedback from students with learning disabilities who have explained that the modularization of content on VetCloud helped them clearly understand what needed to be taken away from each lecture, and breaking down concepts into small chunks meant they did not ‘zone out’. In contrast, general feedback from veterinary science students across year levels at The University of Queensland over the past few years has indicated a strong collective dislike for being provided with only short, modularized videos. Indeed, our VETS1003 students did not find the modularized, short topic videos to be beneficial to their learning until they were offered in conjunction with complete full-length lecture recordings. This combination of modularized, short topic videos with the slower-paced explanations provided in full-length lectures were required by our first-year students for them to perceive that they had been provided with sufficient learning materials to meet their learning needs.

In the flipped classroom approach employed in VETS1003 in 2022, which involved the progressive provision of modularized content on the VetCloud platform, the majority of students felt they were not provided with sufficient learning materials, their work/study/life balance and stress levels were not supported, their use of study time was inefficient, and they did not find the employed approach to be more flexible or convenient than traditional, didactic teaching. Students thus asked for changes to the types and provision time of the offered online learning resources and the introduction of face-to-face lectures. Following the requested changes being integrated in the 2023 flexible learning approach, students largely felt that they were provided with sufficient learning materials and that the employed approach was more flexible and convenient for them, appreciating both having the online resources available to them earlier in the semester and the option to attend face-to-face lectures and/or watch the video recordings of these as it suited them best. The 2023 students reported that their work/study/life balance was supported, stress levels were reduced, and their study time was more efficiently used. This outcome highlights the positive impacts of applying iterative student feedback in teaching, and the importance of considering student perceptions and the student experience when implementing educational interventions.

## 5. Conclusions

Flexible teaching where online learning resources are offered in addition to in-class instruction can be successfully applied to teaching veterinary anatomy and physiology. Applying iterative student feedback regarding the provision of multi-modal learning resources and how they were utilized in teaching across flipped classroom and flexible teaching approaches had a positive impact on veterinary students’ learning experience. In 2023, the use of VetCloud in the flexible learning approach improved students’ work/study/life balance, reduced their stress levels, and enabled them to use their time much more efficiently when studying, compared to the flipped classroom approach used in 2022. This teaching method offers veterinary educators an innovative and efficient approach to veterinary student education in anatomy and physiology.

## Data Availability

The data presented in this study are available in the article.

## Supplementary Materials

The following supporting information can be downloaded at: https://www.mdpi.com/article/10.3390/ani14162335/s1, Supplementary File S1: 2022 Student survey; Supplementary File S2: 2023 Student survey.
