# Supplementary material for: Applying Iterative Student Feedback across Flipped Classroom and Flexible Teaching Approaches: Impact on Veterinary Students’ Learning Experience
Source: Animals (Basel). 2024 Aug 13;14(16):2335. doi: 10.3390/ani14162335 (PMC11350684; doi:10.3390/ani14162335)
Supplement: Supplementary file 1 [file animals-14-02335-s001.zip › Singh et al, Animals, 2024 - Supplementary File S1 - 2022 Survey.pdf]

Supplementary file S1: 2022 survey for VETS1003 students

1. *[Likert scale rating question]* How would you rate the flipped classroom approach using the VetCloud platform for providing convenience and flexibility in your learning, in comparison to traditional course delivery (didactic lectures)?

   Rating scale for responses: 1–5, where:
   - *1* = ‘very poor’ or ‘much less convenient and flexible’
   - *2* = ‘poor’ or ‘less convenient and flexible’
   - *3* = ‘satisfactory’ or ‘same convenience and flexibility’
   - *4* = ‘good’ or ‘more convenient and flexible’
   - *5* = ‘excellent’ or ‘much more convenient and flexible’

2. *[Likert scale rating question]* How would you rate the flipped classroom approach using the VetCloud platform, in comparison to typical pre-recorded lectures?

   Rating scale for responses: 1–5, where:
   - *1* = ‘very poor’
   - *2* = ‘poor’
   - *3* = ‘satisfactory’
   - *4* = ‘good’
   - *5* = ‘excellent’

3. *[Multiple-choice question with one response allowed]* Which format would you prefer to have lecture content delivered in?

   Answer options:
   - Face-to-face lectures
   - Pre-recorded full-length (50-minute) lecture videos
   - Flipped classroom with the VetCloud platform
   - Other

4. *[Open-response question with free textbox]* *[Only offered via question branching to survey respondents who answered question 3 with ‘other’]* If you answered the previous question with ‘other’, what other format would you prefer to have lecture content delivered in?

5. *[Likert scale rating question]* How would you rate the organisation and flexibility of the VetCloud platform for supporting your work/study/life balance?

   Rating scale for responses: 1–5, where:
   - *1* = ‘very poor’
   - *2* = ‘poor’
   - *3* = ‘neutral’
   - *4* = ‘good’
   - *5* = ‘excellent’

6. *[Likert scale rating question]* Do you feel that the flipped classroom approach (modularised content provided on the VetCloud platform, weekly face-to-face tutorial sessions, practical sessions) provides you with adequate support from your lecturer and tutoring team?

   Rating scale for responses: 1–5, where:
   - *1* = 'strongly disagree'
   - *2* = 'disagree'
   - *3* = 'neither agree nor disagree'
   - *4* = 'agree'
   - *5* = 'strongly agree'

7. *[Likert scale rating question]* Do you feel more confident in asking your lecturer and tutoring team questions with the flipped classroom approach than with traditional learning?

   Rating scale for responses: 1–5, where:
   - *1* = 'much less confident'
   - *2* = 'less confident'
   - *3* = 'neither more nor less confident'
   - *4* = 'more confident'
   - *5* = 'much more confident'

8. *[Multiple-choice question with one response allowed]* Do you generally engage with the content before, during, or after the scheduled lecture time in the timetable?

   Answer options:
   - Before
   - During
   - After

9. *[Likert scale rating question]* How do you feel about having allocated time to engage and learn core content BEFORE having face-to-face time in the tutorials/practicals?

   Rating scale for responses: 1–6, where:
   - *1* = 'very unhappy'
   - *2* = 'unhappy'
   - *3* = 'neutral to unhappy'
   - *4* = 'neutral to happy'
   - *5* = 'happy'
   - *6* = 'very happy'

10. *[Multiple-choice question with one response allowed]* On average, how many hours do you engage with the lecture content a week?

    Answer options:
    - 0–1 hour
    - 1–2 hours
    - 2–3 hours
    - 3–4 hours
    - 4–5 hours
    - 5+ hours

11. *[Multiple-choice question with one response allowed]* Do you feel that you are using your time more efficiently learning in this format, compared to didactic teaching (traditional lectures)?

    Answer options:
    - Strongly agree
    - Agree
    - Neither agree nor disagree
    - Disagree
    - Strongly disagree

12. *[Multiple-choice question with one response allowed]* Do you feel that the learning outcomes expected for each module are clear, and provide an understanding of what will be examined from each module?

    Answer options:
    - Strongly disagree
    - Disagree
    - Neither agree nor disagree
    - Agree
    - Strongly agree

13. *[Multiple-choice question with one response allowed]* Do you feel that the flipped classroom approach using VetCloud has reduced your stress levels while learning the content throughout the semester?

    Answer options:
    - Strongly agree
    - Agree
    - Neither agree nor disagree
    - Disagree
    - Strongly disagree

14. *[Likert scale rating question]* How often do you attend the tutorial sessions, either in person or over Zoom?

    Rating scale for responses: 1–5, where:
    - *1* = 'never'
    - *2* = 'less than half the time'
    - *3* = 'half the time',
    - *4* = 'more than half the time'
    - *5* = 'always'

15. *[Multiple-choice question with one response allowed]* How do you generally engage with the weekly tutorial?

    Answer options:
    - Live in person
    - Live online via Zoom
    - Via watching recordings
    - I do not engage with tutorials

16. *[Open-response question with free textbox] [Only offered via question branching to survey respondents who answered question 15 with 'I do not engage with tutorials']* If you answered that you do not engage with tutorials in the previous question, why do you not engage with them?

17. *[Likert scale rating question]* Do you feel that the tutorial sessions are a useful opportunity to consolidate your understanding of concepts and ask questions?

    Rating scale for responses: 1–5, where:
    - *1* = 'not useful at all'
    - *2* = 'not useful'
    - *3* = 'neither useful nor not useful'
    - *4* = 'useful'
    - *5* = 'very useful'

18. *[Open-response question with free textbox]* If you have questions, how and where would you prefer to ask them (*e.g.,* in tutorials)?

19. *[Multiple-choice question with one response allowed]* How would you prefer the tutorials to be delivered?

    Answer options:
    - Face-to-face
    - Online via Zoom
    - Both face-to-face and online via Zoom

20. *[Multiple-choice question with one response allowed]* Do you feel that the practicals help consolidate your learning?

    Answer options:
    - Yes
    - No

21. *[Open-response question with free textbox] [Only offered via question branching to survey respondents who answered question 20 with 'No']* If you answered 'no' in the previous question, why not?

22. *[Multiple-choice question with one response allowed]* Does VetCloud help you prepare for practical classes?

    Answer options:
    - Yes
    - No

23. *[Open-response question with free textbox] [Only offered via question branching to survey respondents who answered question 22 with 'No']* If you answered 'no' in the previous question, why not?

24. *[Multiple-choice question with one response allowed]* How much do you agree with the following statement? “I have sufficient time to complete the practicals.”

    Answer options:
    - Strongly agree
    - Agree
    - Neither agree nor disagree
    - Disagree
    - Strongly disagree

25. *[Multiple-choice question with one response allowed]* How much do you agree with the following statement? “The content of the practicals links well to the lecture content.”

    Answer options:
    - Strongly agree
    - Agree
    - Neither agree nor disagree
    - Disagree
    - Strongly disagree

26. *[Multiple-choice question with one response allowed]* Currently, the lectures are scheduled for Friday at 8–11 am, with the tutorial at 12–1 pm, and the practical class at 1–5 pm. Would you prefer the lectures to be scheduled earlier in the week?

    Answer options:
    - Yes, I would prefer lectures to be scheduled earlier in the week
    - No, I don’t mind tutorials being scheduled on the same day as lectures

27. *[Multiple-choice question with one response allowed]* Do you feel that the flipped classroom approach has provided sufficient learning materials to give you confidence that you understand the content, as outlined by the learning outcomes for each module?

    Answer options:
    - Strongly agree
    - Agree
    - Neither agree nor disagree
    - Disagree
    - Strongly disagree

28. *[Multiple-choice question with one response allowed]* Do you feel that the lecture slides with the transcript is a valuable learning resource?

    Answer options:
    - Strongly agree
    - Agree
    - Neither agree nor disagree
    - Disagree
    - Strongly disagree

29. *[Open-response question with free textbox]* Are there any additional learning materials that you feel would support your learning?

30. *[Likert scale rating question]* Do you find the ‘check your understanding’ quiz questions helpful?

    Rating scale for responses: 1–6, where:
    - *1* = ‘very unhelpful’
    - *2* = ‘unhelpful’
    - *3* = ‘satisfactory to unhelpful’
    - *4* = ‘satisfactory to helpful’
    - *5* = ‘helpful’
    - *6* = ‘very helpful’

31. *[Likert scale rating question]* How confident do you feel that the delivery style of the content on the new VetCloud learning platform has supported you to learn assumed knowledge required in future years of the degree, and your profession?

    Rating scale for responses: 1–6, where:
    - *1* = ‘not at all confident’
    - *2* = ‘not confident’
    - *3* = ‘neutral to not confident’
    - *4* = ‘neutral to confident’
    - *5* = ‘confident’
    - *6* = ‘very confident’

32. *[Open-response question with free textbox]* What did you like/dislike about the VetCloud learning platform? Are there any improvements that you would suggest?

33. *[Open-response question with free textbox]* Do you have any further comments, or feedback on the course thus far?
