# Supplementary material for: Applying Iterative Student Feedback across Flipped Classroom and Flexible Teaching Approaches: Impact on Veterinary Students’ Learning Experience
Source: Animals (Basel). 2024 Aug 13;14(16):2335. doi: 10.3390/ani14162335 (PMC11350684; doi:10.3390/ani14162335)
Supplement: Supplementary file 1 [file animals-14-02335-s001.zip › Singh et al, Animals, 2024 - Supplementary File S2 - 2023 Survey.pdf]

# Supplementary file S2: 2023 survey for VETS1003 students

1. *[Multiple-choice question with multiple responses allowed]* Which **format(s)** do you prefer to have **lecture content** delivered in? *(Select all that apply.)*

   *Prompt: If you select 'Other', please specify by typing an explanation into the textbox.*

   *Answer options:*
   - Face-to-face lectures
   - Pre-recorded full-length (50-minute) lecture videos
   - Short topic videos, as provided on VetCloud
   - Other *[with free textbox]*

2. *[Multiple-choice question with one response allowed]* Do you attend the timetabled **face-to-face VETS1003 lectures**?

   *Answer options:*
   - Yes, always
   - Yes, sometimes
   - No, never

3. *[Multiple-choice question with multiple responses allowed]* What factors influence your decision to attend or not attend face-to-face lectures? *(Select all that apply.)*

   *Prompt: If you select 'Other', please specify by typing an explanation into the textbox.*

   *Answer options:*
   - Commute to Gatton campus
   - Timing/scheduling of face-to-face classes
   - Preference for studying independently (*e.g.,* using VetCloud)
   - Work (*e.g.,* timing and availability of shifts)
   - Caring responsibilities
   - Other *[with free textbox]*

4. *[Multiple-choice question with multiple responses allowed]* If you do not attend the face-to-face lectures or miss one/some, **what are you usually doing during the timetabled class time(s)**? *(Select all that apply.)*

   *Prompt: If you select 'Other', please specify by typing an explanation into the textbox.*

   *Answer options:*
   - Self-directed study
   - Working
   - Caring responsibilities
   - Other *[with free textbox]*

5. *[Multiple-choice question with multiple responses allowed]* When do you generally engage with the content on VetCloud – before, during, or after the scheduled lecture time in the timetable? *(Select all that apply.)*

   *Answer options:*
   - Before
   - During
   - After

6. *[Multiple-choice question with multiple responses allowed]* Which of the resources available on VetCloud do you engage with? *(Select all that apply.)*

   *Answer options:*
   - Complete lecture recording videos
   - Short topic videos
   - Learning outcomes for lectures/topics
   - Lecture slides PDF documents
   - Lecture slides with transcript PDF documents
   - ‘Check your understanding’ quiz questions

7. *[Multiple-choice question with one response allowed]* Which of the resources available on VetCloud do you **typically engage with first** when studying the content for **lectures that you <u>have not</u> attended in person**?

   *Answer options:*
   - Complete lecture recording videos
   - Short topic videos
   - Learning outcomes for lectures/topics
   - Lecture slides PDF documents
   - Lecture slides with transcript PDF documents
   - ‘Check your understanding’ quiz questions

8. *[Multiple-choice question with one response allowed]* Which of the resources available on VetCloud do you **typically engage with first** when revising the content for **lectures that you <u>have</u> attended in person**?

   *Answer options:*
   - Complete lecture recording videos
   - Short topic videos
   - Learning outcomes for lectures/topics
   - Lecture slides PDF documents
   - Lecture slides with transcript PDF documents
   - ‘Check your understanding’ quiz questions

9. *[Likert scale rating question]* On a scale of 1–5, how do you rate the **video resources provided on VetCloud** in comparison to typical pre-recorded lectures that may be provided on Blackboard?

   *Rating scale for responses: 1–5*, where:
   - *1* = 'very poor'
   - *2* = 'poor'
   - *3* = 'acceptable' or 'satisfactory'
   - *4* = 'good'
   - *5* = 'excellent'

10. *[Open-response question with free textbox]* **How and when** do you engage with the **videos on VetCloud** when studying the content for **lectures that you have not attended in person**? What videos do you **engage with first**? Please explain.

    *Prompt:*
    *You may find these guiding questions helpful when explaining how and when you interact with the videos:*
    - *Do you watch the complete lecture recording first and then watch the short topic videos after that?*
    - *Do you watch the short topic videos first and then watch the complete lecture recording video later?*
    - *Do you watch only the complete lecture recording videos?*
    - *Do you watch only the short topic videos?*

11. *[Open-response question with free textbox]* **How and when** do you engage with the **videos on VetCloud** when revising the content for **lectures that you have attended in person**? What videos do you **engage with first**? Please explain.

    *Prompt:*
    *You may find these guiding questions helpful when explaining how and when you interact with the videos:*
    - *Do you watch the complete lecture recording videos at all when revising after attending the face-to-face lectures?*
    - *Do you watch only the short topic videos for revision?*
    - *Do you watch both the complete lecture and short topic videos when revising? If so, which do you watch first?*

12. *[Open-response question with free textbox]* **How and when** do you engage with the **lecture slides PDF documents** and/or **lecture slides with transcript PDF documents provided on VetCloud** for [Lecturer's] VETS1003 lectures? Please explain.

    *Prompt:*
    *You may find these guiding questions helpful when explaining how and when you interact with the lecture slides PDF documents:*
    - *Do you look at the lecture slides PDF documents on VetCloud at all or only on Blackboard? When do you look at them?*
    - *Do you look at the lecture slides with transcript PDF files on VetCloud at all? When do you look at them?*

13. *[Likert scale rating question]* On a scale of 1–5, how much do you agree with the following statement? "**The learning outcomes provided on VetCloud for [Lecturer's] VETS1003 lectures are clear and provide an understanding of what will be examined from each lecture.**"

    *Rating scale for responses: 1–5*, where:
    - *1* = 'strongly disagree'
    - *2* = 'disagree'
    - *3* = 'neither agree nor disagree'
    - *4* = 'agree'
    - *5* = 'strongly agree'

14. *[Open-response question with free textbox]* **How and when** do you engage with the **learning outcomes provided on VetCloud** to guide your study for [Lecturer's] VETS1003 lectures? Please explain.

    *Prompt:*
    *You may find these guiding questions helpful when explaining how and when you interact with the learning outcomes:*
    - *Do you read the learning outcomes at all?*
    - *Do you read the learning outcomes before attending face-to-face lectures?*
    - *Do you read the learning outcomes and then watch the videos?*
    - *Do you use the learning outcomes for the short topic videos to help you find which videos will help you revise particular content/concepts?*
    - *Do you watch the videos first when studying the content, and then read the learning outcomes afterwards when revising?*

15. *[Likert scale rating question]* On a scale of 1–5, how much do you agree with the following statement? "**I find the 'check your understanding' quiz questions helpful.**"

    *Rating scale for responses: 1–5*, where:
    - *1* = 'strongly disagree'
    - *2* = 'disagree'
    - *3* = 'neither agree nor disagree'
    - *4* = 'agree'
    - *5* = 'strongly agree'

16. *[Open-response question with free textbox]* **How and when** do you engage with the **quiz questions on VetCloud**? Please explain.

    *Prompt:*
    *You may find these guiding questions helpful when explaining how and when you interact with the quiz questions:*
    - *Do you attempt quizzes after attending face-to-face lectures to test your understanding before you watch any videos on VetCloud?*
    - *Where these are available, do you attempt the quizzes for each short topic video after watching the relevant video?*
    - *Do you attempt the quizzes after interacting with all the videos for a particular lecture or lecture series?*
    - *Do you engage with the quizzes regularly during semester or mostly when revising leading up to assessments?*

17. *[Multiple-choice question with multiple responses allowed]* If you have questions regarding lecture content while working through the resources on VetCloud, what is your preferred way to ask these? *(Select all that apply.)*

    *Prompt: If you select 'Other', please specify by typing an explanation into the textbox.*

    *Answer options:*
    - Email
    - Ask a question during lectures
    - Ask a question during practicals/tutorials
    - Discussion board
    - Other *[with free textbox]*

18. *[Multiple-choice question with one response allowed]* On average, for how many hours do you engage with the content for **each lecture on VetCloud** **in the week that it is timetabled**?

    *Answer options:*
    - 0–1 hour
    - 1–2 hours
    - 2–3 hours
    - 3–4 hours
    - 4–5 hours
    - 5+ hours

19. *[Multiple-choice question with one response allowed]* On average, for how many hours do you engage with the content for **each lecture on VetCloud** **in total, including revision for assessments**?

    *Answer options:*
    - 0–1 hour
    - 1–2 hours
    - 2–3 hours
    - 3–4 hours
    - 4–5 hours
    - 5+ hours

20. *[Multiple-choice question with one response allowed]* On average, for how many hours do you engage with **VETS1003 content** in total **each week**?

    *Prompt: This question is asking about **all the time you spend interacting with any and all VETS1003 content/resources**, including resources on VetCloud, Blackboard, Slide Box, practicals, tutorials, face-to-face lectures, revision and self-directed study time, etc.)*

    *Answer options:*
    - 0–1 hour
    - 1–5 hours
    - 6–10 hours
    - 10+ hours

21. *[Likert scale rating question]* On a scale of 1–5, how much do you agree with the following statement? "**VetCloud provides access to sufficient learning materials to support my learning in VETS1003.**"

    *Rating scale for responses: 1–5*, where:
    - *1* = 'strongly disagree'
    - *2* = 'disagree'
    - *3* = 'neither agree nor disagree'
    - *4* = 'agree'
    - *5* = 'strongly agree'

22. *[Open-response question with free textbox]* Are there any **additional learning materials** that you feel would help to support your learning? Please explain.

23. *[Likert scale rating question]* On a scale of 1–5, how do you rate the **VetCloud platform** for providing **convenience and flexibility in your learning**, in comparison to traditional course delivery (didactic lectures delivered at set times)?

    *Rating scale for responses: 1–5*, where:
    - *1* = 'much less convenient and flexible than didactic lectures'
    - *2* = 'less convenient and flexible than didactic lectures'
    - *3* = 'same convenience and flexibility as didactic lectures'
    - *4* = 'more convenient and flexible than didactic lectures'
    - *5* = 'much more convenient and flexible than didactic lectures'

24. *[Likert scale rating question]* On a scale of 1–5, how do you rate the **VetCloud platform** for supporting your **work/study/life balance**?

    *Rating scale for responses: 1–5*, where:
    - *1* = 'very poor'
    - *2* = 'poor'
    - *3* = 'neutral
    - *4* = 'good'
    - *5* = 'excellent'

25. *[Likert scale rating question]* On a scale of 1–5, how much do you agree with the following statement? "**I use my time more efficiently when learning using VetCloud, compared to didactic teaching (traditional lectures).**"

    *Rating scale for responses: 1–5*, where:
    - *1* = 'strongly disagree'
    - *2* = 'disagree'
    - *3* = 'neither agree nor disagree'
    - *4* = 'agree'
    - *5* = 'strongly agree'

26. *[Likert scale rating question]* On a scale of 1–5, how much do you agree with the following statement? “**Having access to VetCloud as a learning resource has reduced/is reducing my stress levels during the semester, including in the lead up to assessments.**”

    *Rating scale for responses: 1–5*, where:
    - *1* = ‘strongly disagree’
    - *2* = ‘disagree’
    - *3* = ‘neither agree nor disagree’
    - *4* = ‘agree’
    - *5* = ‘strongly agree’

27. *[Likert scale rating question]* On a scale of 1–5, how much do you agree with the following statement? “**I feel confident that VetCloud has supported me to learn the assumed knowledge required in future years of my degree, and my profession.**”

    *Rating scale for responses: 1–5*, where:
    - *1* = ‘strongly disagree’
    - *2* = ‘disagree’
    - *3* = ‘neither agree nor disagree’
    - *4* = ‘agree’
    - *5* = ‘strongly agree’

28. *[Multiple-choice question with one response allowed]* Do you feel that you receive adequate **support from your lecturers and tutoring team** in VETS1003, particularly if you primarily use VetCloud to engage with lecture content?

    *Answer options:*
    - Yes
    - Yes, somewhat
    - No

29. *[Open-response question with free textbox]* What **additional support** do you feel would improve your learning experience in VETS1003? Please explain.

30. *[Open-response question with free textbox]* What do you **like** about the **VetCloud platform**? Please explain.

31. *[Open-response question with free textbox]* What do you **dislike** about the **VetCloud platform**? Do you have any **suggestions for improvement**? Please explain.

32. *[Open-response question with free textbox]* Do you have any **other comments** about the **VetCloud platform** and/or how it has been used in VETS1003?
